# Electron Beam Powder Bed Fusion of Water Atomized Iron and Powder Blends

**DOI:** 10.3390/ma15041567

**Published:** 2022-02-19

**Authors:** Alexander Kirchner, Burghardt Klöden, Marie Franke-Jurisch, Gunnar Walther, Thomas Weißgärber

**Affiliations:** Fraunhofer Institute for Manufacturing Technology and Advanced Materials IFAM, 01277 Dresden, Germany; burghardt.kloeden@ifam-dd.fraunhofer.de (B.K.); marie.franke-jurisch@ifam-dd.fraunhofer.de (M.F.-J.); gunnar.walther@ifam-dd.fraunhofer.de (G.W.); thomas.weissgaerber@ifam-dd.fraunhofer.de (T.W.)

**Keywords:** additive manufacturing, electron beam powder bed fusion, water atomization, iron, powder blends

## Abstract

In the present state of the art, highly spherical alloy powders are employed as feedstock in powder bed fusion processes. These powders are characterized by high flowability and apparent density. Their elaborate fabrication process is reflected in high powder price, adding a significant fraction to the cost of additively manufactured parts. Thus, the use of non-spherical powders, such as water atomized material, can lower costs significantly. Here, the electron beam powder bed fusion (PBF-EB) of standard water atomized iron powder used for press-and-sinter is studied. Despite raking problems, using the coating mechanism in standard configuration samples with densities exceeding 99% were fabricated. In a further step, the addition of alloying elements by powder blending is explored. Important powder properties of feedstock blended from irregular and spherical powders are characterized. The PBF-EB processing of two alloys is presented. The first represents a low carbon steel. Samples were characterized by metallographic cross-section, energy dispersive X-ray (EDX) mapping, and mechanical testing. The second alloy system is a FeCrAl. After PBF-EB processing of the powder mixture, chemical homogeneity was achieved. Besides the low cost, this approach of using water atomized powder mixed with master alloy offers the advantage of high flexibility for potential application.

## 1. Introduction

In current commercial powder bed fusion (PBF) processes, the use of powders with spherical morphology is prevalent. The reason is their beneficial behavior. High flowability facilitates powder feeding and the deposition of layers with uniform density. Processes capable of fabricating spherical particle morphology are several types of gas atomization, plasma atomization, and plasma rotating electrode process [1]. The relatively low solidification rate in these processes leads to highly spherical particles. In addition, high chemical purity can be achieved. The consumption of high purity process gas and a low yield in the target fraction are reasons for the high prices of metal additive manufacturing (AM) feedstock. An alternative for materials with low reactivity but at far lower cost is water atomization (WA). Due to the higher solidification rate, the manufactured powder is characterized by irregular shape with a coarse surface topology, a wide size distribution, and lower flowability.

Motivated by that reasoning, several investigations of laser-based powder bed fusion (PBF-LB) of WA iron-based powders have been performed. Letenneur et al. used fine fractions of water atomized iron powder [2]. At volumetric energies exceeding 50 J/mm^3^, they were able to fabricate specimen with 99.5% density. The best samples achieved an ultimate tensile strength of 330 MPa, clearly surpassing the properties of sintered material. Rogalsky et al. noted that the PBF-LB processing of WA iron powder required modification to the powder feeding mechanism [3]. The powder bed density was found sufficiently uniform for AM. Li and coworkers processed water atomized 316L stainless steel powder [4]. Due to the occurrence of lack of fusion defects and micro cracks, they found the result inferior to gas atomized powder. Haferkamp et al. compared part density from 316L powders of different circularity [5]. WA powder yielded higher porosity than more spherical powders. Pasebani et al. studied different 17-4 PH stainless steel powders for PBF-LB [6]. They found differences in the as-built microstructure that warrant different heat treatment schedules. Several studies on the PBF-LB of AISI 4130 low alloy steel powder prepared by WA have been conducted [7,8]. These demonstrate the feasibility of AM processing of WA powder. Some differences to gas atomized powders were noted. The cross-sectional area of melt tracks is smaller in WA powder due to lower packing density. The amount of ejected spatter is larger, and SiO_2_ inclusions were observed. Both effects are attributed to higher oxygen content.

For AM of alloys by PBF, a pre-alloyed feedstock is employed almost exclusively. That way, the formation of a homogenous alloy does not have to take place during the very short time the material exists in the liquid state. On the other hand, there are motives for processing blends of different powders. Tailoring alloys from varying contents of base powders offers higher flexibility by avoiding custom atomization. Furthermore, not all materials are equally suitable for all powder atomization processes. Processing powder blends may result in technological or economic advantages. In addition, starting from a chemically heterogeneous state offers the potential to create non-equilibrium microstructures. For those reasons, a growing number of studies deal with PBF of powder blends.

Song et al. investigated the PBF-LB processing of Fe and SiC powders [9]. Nanosized SiC particles in martensitic and pearlitic matrices were obtained. An ultimate tensile strength of 753 MPa was measured. AlMangour et al. reported the mixing and milling of 316L powder with TiB2 and subsequent densification [10]. Hot isostatic pressing was effective in reducing internal porosity and cracks but resulted in coarsening of the reinforcement particles. Köhler et al. added Cr_3_C_2_ to AISI H13 tool steel powder before PBF-LB [11]. While segregation tendency was noted, this led to a reduction of crack density. Köhnen et al. employed powder blends for fast alloy screening in PBF-LB manufactured steel [12]. The same approach was taken in the experimental development of high-entropy alloys [13]. Finally, Huber et al. published a study on in-situ alloy formation of high-melting W, Ta, Mo, and Nb particles in a Ti-matrix [14]. Scan parameter combinations beneficial to chemical homogeneity were identified.

Powder blends have been processed by PBF-EB as well. One advantage of the PBF-EB technology for studies of non-conventional powders is that relatively thick powder layers can be processed. Koptyug et al. described the PBF-EB of powder blends of well flowing gas atomized powders with fine and irregular shaped material [15]. Depending on the process parameters, they achieved in-situ alloying or fabricated unconventional microstructures. Momeni et al. reported the PBF-EB processing of dendritic copper powder mixed with blocky chromium particles [16]. Here, only the copper component was fully molten. Popov et al. used elemental powder blends to synthesize the high-entropy alloy Al0.5CrMoNbTa0.5 [17]. Hankwitz et al. fabricated niobium-tungsten-zirconium alloys from blends of plasma spheroidized powders [18]. The as-built microstructure is still inhomogeneous, but post-processing heat treatments can mitigate the defects.

The scope of this study addresses several questions concerning the use of non-standard powder in AM: (1) Is the PBF-EB processing of WA iron powder used in conventional press-and-sinter powder metallurgy feasible? In contrast to [2,3], the coarse powder fraction is also used. (2) How does mixing non-spherical WA powder with spherical powder improve its process ability in terms of flowability and apparent density? (3) Is blending WA iron powder a possible route for PBF-EB of alloys? This topic is approached twofold. In a first test, WA iron is mixed with relatively small amounts of spherical 316L powder and graphite powder to yield a model alloy. Using 10 wt.% 316L and 0.25 wt.% graphite, a nominal composition of 1.8 wt.% Cr, 1.3 wt.% Ni, 0.2 wt.% Mo, and 0.25 wt.% C results. This is comparable to the tempering steel grades 25CrMo4 and 34CrNiMo6. In a second test, WA iron is blended with an equal amount of CrAl-master alloy to create FeCrAl. The aim here is to achieve an Al-content well above 5 wt% for increased high-temperature corrosion resistance.

## 2. Materials and Methods

Water atomized iron powder designated ASC100.29 with an approximate particle size from 20 µm to 180 µm was supplied by Höganäs Sweden AB (Höganäs, Sweden). Spherical 316L powder with a nominal particle size from 45 µm to 106 µm was obtained from Carpenter Powder Products AB (Torshälla, Sweden). Flake graphite with a minimum of 95% carbon and a particle size below 45 µm was supplied by NGS Naturgraphit Leinburg. Fe-34.9Cr-15.9Al with a particle size below 53 µm was provided by H.C. Starck GmbH Laufenburg, Germany. Where applicable mixing was performed, a Turbula shaker mixer was used (Glen Mills, Clifton, NJ, USA). Flowability was characterized for 50 g of powder by means of a calibrated funnel with 2.54 mm diameter (Hall flowmeter, DIN ISO 4490). Apparent density was determined by the funnel method (DIN ISO 3923/1).

PBF-EB was performed on an Arcam A2X machine (Arcam AB, Gothenburg, Sweden) using software version EBM Control 3.2. Before starting a build process, the chamber was evacuated to at least 1 × 10^−4^ mbar. Afterwards, helium pressure of 2 × 10^−3^ mbar was employed. The acceleration voltage of the electron gun was 60 kV. To minimize the necessary amount of powder, a custom-made build tank of 100 mm × 100 mm size was used. It employed the rake system in its standard configuration. The main difference in comparison to standard-sized build space are the values of the preheat parameters. For parameter tests, cubes of 15 mm edge length were fabricated. To compare different parameter sets, the volumetric energy density E_V_, defined as

(1) $$E_{V}=\frac{P}{v\cdot h\cdot t},$$

has proven useful. It is the ratio between beam power *P*, scan speed *v*, hatch distance *h*, and layer thickness *t*. At the beginning of a build process, a steel start plate 80 mm square was heated to 750 °C as measured by a thermocouple underneath. The addition of each layer started by raking a powder layer of 100 μm nominal thickness. The fresh powder was preheated by fast scanning with a defocused electron beam. Then, the specimens were solidified by scanning the surface at a constant line offset (lateral distance between parallel lines) of 100 μm with a focused electron beam (focus offset 3 mA). Between layers, the principal scan direction was alternated from X-axis to Y-axis. Upon completion, the build was cooled slowly inside the process chamber. Excess powder was removed by nitrogen jet blasting. For recycling, the powder was screened using a 40 mesh/400 µm sieve.

For metallographic preparation, specimens were cut both in and perpendicular to build direction, mounted, and then polished. To reveal the microstructure, polished samples were etched with Nital or V2A etchant. Scanning electron microscopy (SEM) and energy-dispersive X-ray spectroscopy (EDX) were performed on a Zeiss EVO 50 XVP (Carl Zeiss GmbH, Oberkochen, Germany).

The hardness was measured by a Vickers test using an Innovatest Falcon 500 instrument (Innovatest GmbH, Selfkant-Heilder, Germany) according to DIN EN ISO 6507-1. Heat treatment of the WA iron/10 wt.% 316L/0.25 wt.% C consisted of austenitizing at 850 °C for 20 min followed by oil quenching. Tempering was done at 600 °C for 1 h. For tensile tests, flat specimens with 2 mm thickness, 6 mm width, and 26 mm length of the reduced section according to DIN 50125:2016-12 (Type E) were cut from blanks using electrical discharge machining. Tensile tests were performed on a Zwick 1476 machine (ZwickRoell GmbH and Co. KG, Ulm, Germany) at room temperature and a deformation rate of 0.5 mm/min. A minimum of five specimen were tested perpendicular to the build direction.

## 3. Results

### 3.1. Characterization and PBF-EB of Water Atomized Iron Powder

**Figure 1:**
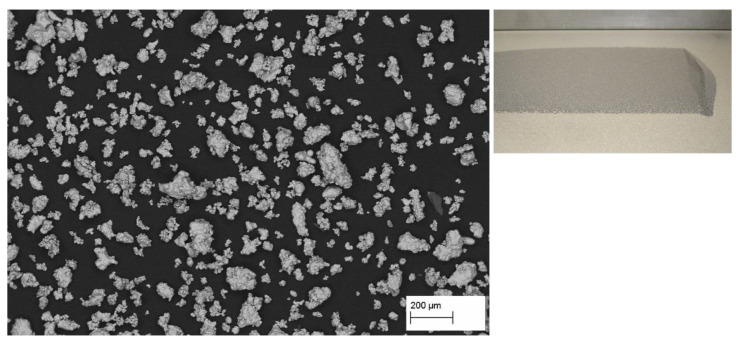
**Left**: Scanning electron micrograph of virgin water atomized ASC100.29 iron powder. **Right**: Test-raked layer with 100 µm thickness.

Figure 1 shows the micrograph of water atomized iron powder. The irregular particle shape is immediately obvious. The particle size ranges roughly from 20 µm to 200 µm. The Hall flow rate is 26.8 s for 50 g, while an apparent density of 3.02 g/cm^3^ was established. Both values agree well with the manufacturer’s information.

With its low flowability and apparent density, the pure water atomized powder represents suboptimal processability in powder bed AM. Therefore, its PBF-EB processing was investigated as a baseline. The choice of layer thickness is influenced by the largest particles present. Raking 100 µm thick layers yielded reasonably uniform deposits. During the build process, the raking process proved stable, and very few “zero pulses” occurred. The preheat parameters were derived from austenitic stainless steels, except that the overall temperature was kept between 700 °C and 750 °C. Using these preheat settings, no process instabilities (smoke events) occurred, and the sintered surplus powder could be removed by jet blasting.

**Figure 2:**
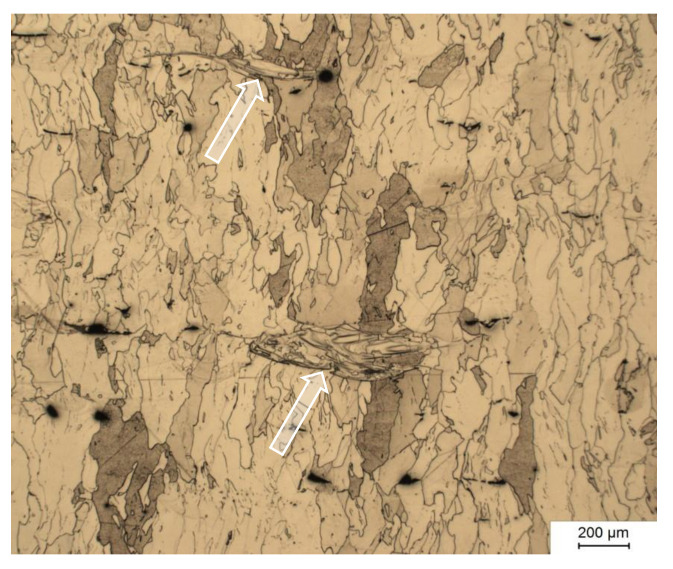
Optical micrograph of PBF-EB processed iron powder (etched using Nital). Build direction is vertical.

For the development of the PBF-EB melt parameters, cubes of 15 mm edge length were fabricated and assessed mainly according to density established from metallographic cross-sections. As in other materials, a minimum E_V_ is necessary for high density. Using beam currents from 3 mA to 6 mA, corresponding to 180 W to 360 W beam power, densities exceeding 99% were found for volumetric energy densities of 50 J/mm^3^ and more. At energy density well below that threshold, the test cubes’ melt surface is visibly porous. Another limit to the process window is given by the formation of uneven melt surfaces, called swelling. This happens at high energy densities and is especially prevalent at high beam power. Specimen with smooth and even melt surface were characterized by metallographic cross-section to analyze the remaining porosity. Optimum parameter sets were chosen due to minimum porosity. Figure 2 shows iron material PBF-EB fabricated with a 4 mA beam current and 400 mm/s scan speed, corresponding to a volumetric energy density of 60 J/mm^3^. The ferrite grains were elongated along the build direction. In some areas marked with arrows, the microstructure differs. There, the grains exhibit divergent aspect ratio, possibly indicating incomplete melting of larger particles. The hardness established for PBF-EB fabricated pure iron is 77 HV5.

**Figure 3:**
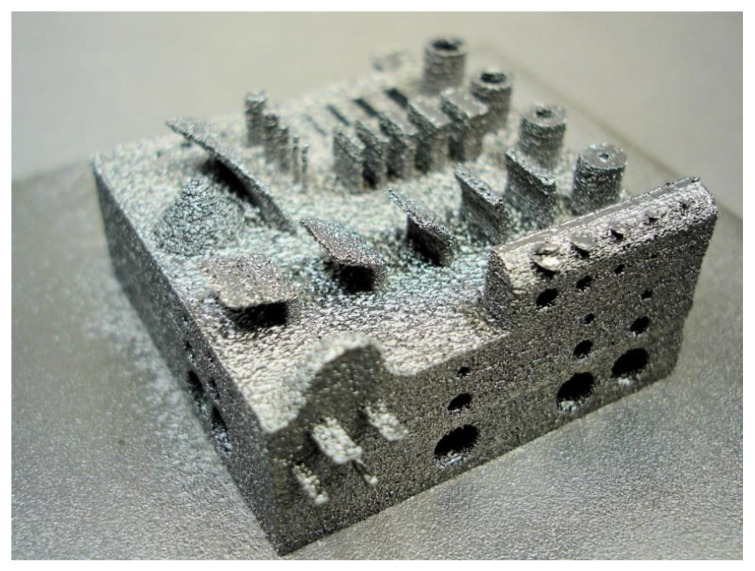
Test geometry manufactured by PBF-EB from ASC100.29 iron. The part size is 40 mm (width) × 40 mm (depth) × 24 mm (height). The design originates from the BMBF supported project AGENT-3D.

Pure water atomized iron powder was recycled up to five times. Sieving with a 400 µm mesh did not remove a significant amount of material. This implies that jet blasting was sufficient to break up agglomerated particles. Based on visual evaluation of the uniformity of raked layers and the absence of process instabilities such as smoke events, the processability of the recycled powder was found identical to virgin powder. Furthermore, first attempts were made to fabricate geometrically complex parts, as shown in Figure 3. Overall, the part surface is characterized by high roughness. In the geometry test, several design elements such as holes, thin walls, cylinders, and unsupported overhangs were successfully realized. The minimum dimensions built were 0.8 mm thickness for vertical walls, 0.7 mm thickness for horizontal walls without support, 0.7 mm diameter for vertical cylinders, 0.8 mm diameter for horizontal cylinders, and 0.7 mm wall thickness for the hollow cylinder. Horizontal holes with diameters ranging from 1 mm to 4 mm were realized. At 4 mm diameter, some deviation from circularity called sagging was observed. It proved very difficult to remove surplus powder from holes and slots of 1 mm dimension or below.

### 3.2. Behavior of Mixtures with Spherical Powders

**Figure 4:**
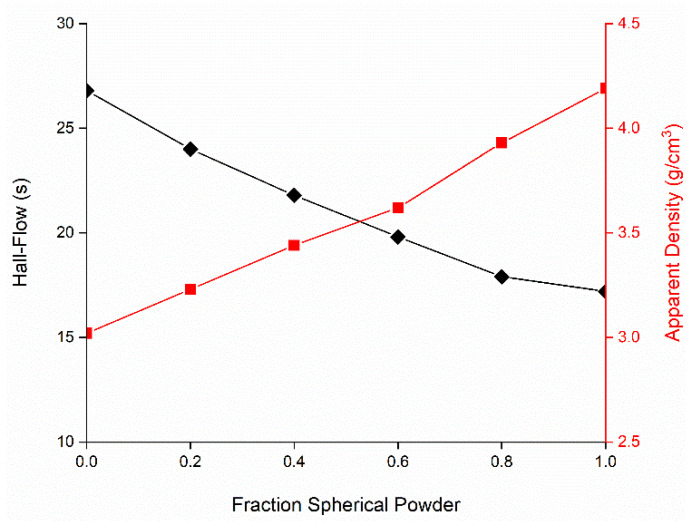
Flowability (**black**) and apparent density (**red**) of powder mixtures of water atomized iron and spherical 316L.

Mainly due to the reduced flowability, the processability of the water atomized powder is inferior to gas atomized material. By blending the irregular shape with spherical powder, the processability can be improved. Figure 4 demonstrates the possible extend of increase in flowability and apparent density. The other end was given by the pure spherical powder with a Hall flow rate of 17.2 s and apparent density of 4.19 g/cm^3^. The properties of mixtures vary nearly linearly with composition.

### 3.3. PBF-EB of WA Iron—316L-C

**Figure 5:**
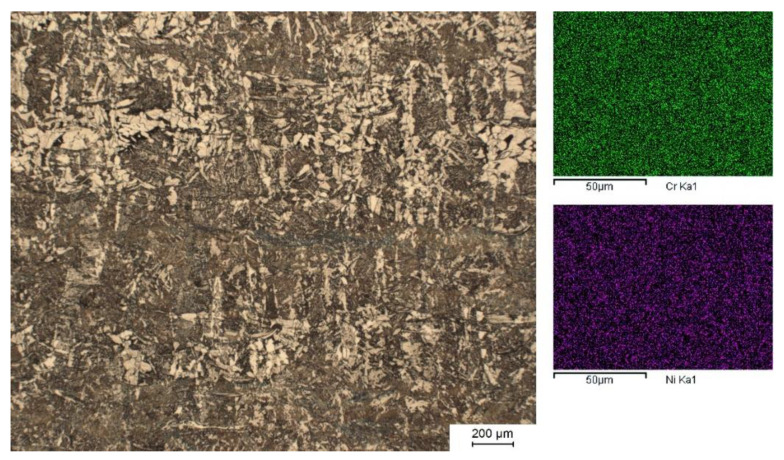
**Left**: Optical micrograph of PBF-EB processed iron/10 wt% 316L/0.25 wt% C (etched with Nital). Build direction is vertical. **Right**: EDX mappings of Cr and Ni.

To investigate the possibility of alloy formation by powder blending, 316L powder was added as a model master alloy. In a subsequent step, carbon was added via graphite powder. For 10 wt.% 316L and 0.25 wt.% C, the same PBF-EB process parameters were applicable as for pure water atomized iron powder. Full density was reached for various parameter sets with E_V_ exceeding 40 J/mm^3^. The specimen shown in Figure 5 was manufactured with 4 mA beam current and 600 mm/s scan speed. The etched cross-section reveals fine grain with some microstructural inhomogeneity apparent. The distribution of ferrite and perlite is layered but at a scale greater than individual powder layers.

EDX measurements confirm the expected presence of the main alloying elements; 2.1 wt.% Cr and 1.6 wt.% Ni were detected for the sample in Figure 5. Furthermore, element mappings demonstrate the homogenous spatial distribution of Cr and Ni.

**Figure 6:**
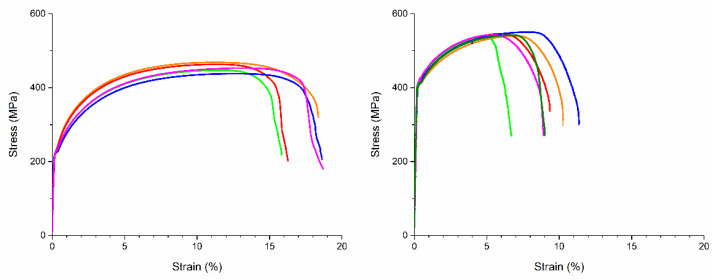
Stress–strain curves of tensile test of PBF-EB processed (**left**) and heat-treated (**right**) iron/10 wt.% 316L/0.25 wt.% C tested perpendicular to build direction.

As a first indication of the mechanical properties, the hardness was measured in the PBF-EB sample with 10 wt.% 316L and 0.25 wt.% C. In the as-built state, a hardness of 272 HV10 was measured. The result of the tensile test in the as-built state is depicted in Figure 6 left. The average yield strength was 231 MPa, the ultimate tensile strength 454 MPa, and elongation at break 17.5% (Table 1). After applying a standard heat-treatment consisting of oil quenching from 850 °C and tempering at 600 °C, the yield strength increased to 416 MPa and the ultimate tensile strength to 543 MPa (Figure 6 right). The ductility of the heat-treated material was lower at 9.0%.

**Figure 7:**
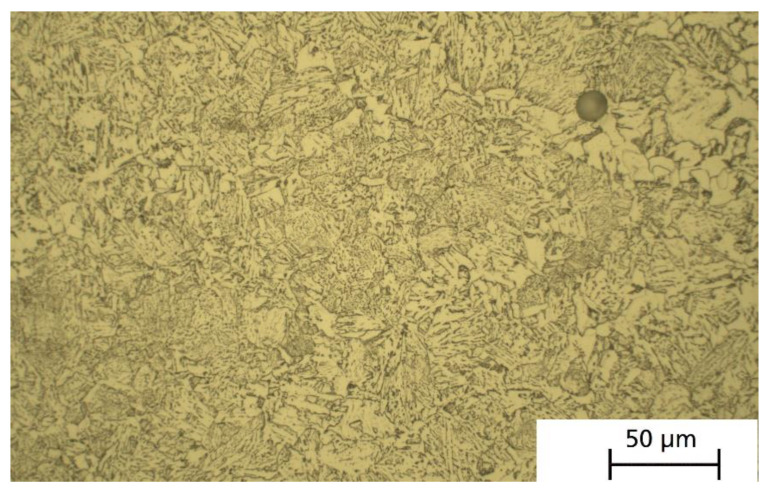
Optical micrograph of PBF-EB processed and heat-treated iron/10 wt.% 316L/0.25 wt.% C (etched with Nital). Build direction is vertical.

Metallographic analysis of the heat-treated specimen at low magnification showed some inhomogeneity again. Similar to Figure 5, they are coarser than individual layers. Figure 7 depicts the microstructure at higher magnification. It consists of ferrite grains and a fine network of precipitated carbides.

### 3.4. PBF-EB of WA Iron—FeCrAl

**Figure 8:**
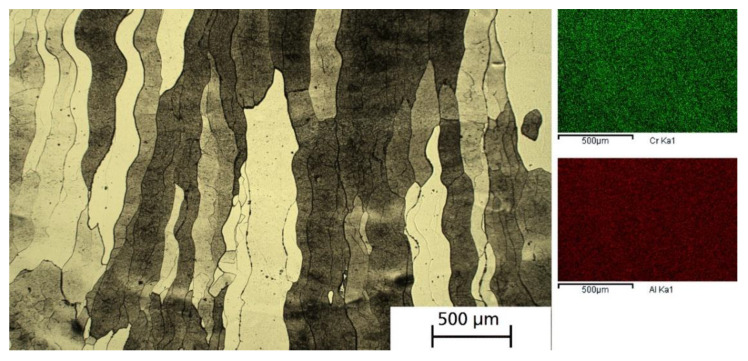
**Left**: Optical micrograph of PBF-EB processed WA iron/50 wt.% FeCrAl (etched with V2A etchant). Build direction is vertical. **Right**: EDX mappings of Cr and Al.

For these experiments, equal parts by weight of WA iron powder and Fe-34.9Cr-15.9Al master alloy were blended. The theoretical chemical composition is Fe-17.5Cr-8Al. Blending increases the flowability and apparent density compared to pure WA iron. The measured Hall flow rate was 23.0 s, and the apparent density was 3.54 g/cm^3^. Due to the fine fraction of the master alloy, some dusting was observed. Because this blend showed less sintering activity compared to previously mentioned iron materials, the preheat temperature of the powder bed had to be increased to 950 °C to provide PBF-EB process stability. During the melt parameter development, it was noted that high beam power and high energy density led to pronounced swelling of the top surface. High densities were obtained for E_V_ in the range of 40 J/mm^3^ to 70 J/mm^3^. The specimen displayed in Figure 8 was PBF-EB-fabricated with 5 mA beam current and 750 mm/s scan speed. Its microstructure is characterized by very large grains elongated in build direction. The chromium content measured by EDX was 18.2 wt.%, while the aluminum content was 7.5 wt.%. Other samples with higher volumetric energy density showed reduced content of alloying elements. A specimen with E_V_ of 60 J/mm^3^ contained only 16.0 wt.% Cr and 6.5 wt.% Al. In all cases, the EDX element mappings showed a homogeneous distribution of Cr and Al on a scale of several 100 µm.

## 4. Discussion

With its irregular shape, wide particle size distribution, low flowability, and low apparent density, WA ASC100.29 iron powder represents a challenge for AM in PBF processes. Employing PBF-EB the fabrication of iron with densities exceeding 99% is demonstrated. The necessary volumetric energy is similar to that required for gas atomized 316L [19]. There, an energy density of 40 J/mm^3^ was found to be the threshold for fabricating dense material from 70 µm layers. Some binding faults remained even after increasing the energy density. PBF-EB of pure WA iron leads to an anisotropic microstructure with ferrite grains of several 100 µm length elongated in build direction. Columnar grain with a preferential <001> orientation in build direction is common in PBF-EB processed materials with a cubic crystal structure [20].

Blending WA powder with spherical powder increases its flowability and apparent density with almost linear correlation. At the same time, powder blending can be used to adjust the alloy composition. In one experiment, WA ASC100.29 iron powder was blended with 10 wt.% 316L and 0.25 wt.% C. This resulted in a chemical composition resembling commercial tempering steel. PBF-EB process parameters identical to pure WA iron are applicable. Some differences are observed in comparison to pure WA iron powder. The achievable porosity is lower for the alloy. The columnar grain disappears, and the microstructure is finer. Some microstructural inhomogeneity is apparent as coarse layering. Despite that, as far as can be detected using EDX, the alloying elements Cr and Ni are distributed homogeneously.

To compare the hardness of the carbon-alloyed material, both composition and heat treatment condition must be taken into account. Low-alloy steel with 0.25 wt.% C with 50% martensite was given as 320 HV [21]. Hardness was reduced to below 200 HV by tempering above 600 °C. The commercial alloy 25CrMo4 has a maximum hardness of 225 HV in annealed condition [22]. The higher alloyed 34CrNiMo6 reached 260 HV in the annealed state [23]. Therefore, the hardness value of 272 HV10 measured on the PBF-EB processed iron/10 wt.% 316L/0.25 wt.% C was plausible for a material with homogenous elemental distribution.

Due to the high temperature of the powder bed during the build process and the slow cooling after build completion, the as-built material corresponded to annealed steel with reduced strength. After quenching and tempering, the tensile strength of the PBF-EB material increased compared to the as-built state. It did not reach the tensile strength and elongation values detailed for heat treated 25CrMo4 and 34CrNiMo6, however. Possible explanations are remaining porosity and the not fully homogeneous microstructure.

The second blend employed equal amounts of WA iron and CrAl master alloy powder to form FeCrAl. Despite using finer powder, both flowability and apparent density roughly adhered to the relation shown in Figure 4. During PBF-EB processing, a distinct swelling tendency was observed for this material at a higher energy density. This correlates with a measured loss of the alloying elements Cr and Al. The highest content established in a dense sample was 18.2 wt.% Cr and 7.5 wt.% Al. The microstructure was columnar and characterized by very large ferrite grains, as both Cr and Al have a strong stabilizing effect on the body-centered cubic structure [24]. Using EDX, a homogeneous spatial distribution of the alloying elements was found. For FeCrAl of the above-mentioned composition, the occurrence of welding-induced cracking in claddings has been reported [25]. In the PBF-EB specimen, no cracks were detected, possibly due to the high process temperature.

For the described experimental work, a custom-made small build tank and only relatively small amounts of powder were used. While for blended powders in large hoppers the possibility of segregation of the components exists, the powder reservoir in the slopes is circulated by the rake. Therefore, it was not investigated here.

## 5. Conclusions

The investigation presented within this paper deals with the use of non-standard powder for additive manufacturing by PBF-EB. The key findings are:

(1) PBF-EB processing of WA iron powder used for press-and-sinter can lead to highly dense material with less than 1% porosity. This is despite the fact that the irregular shape of the WA iron powder causes comparably low flowability and apparent density.
(2) Blending the irregularly shaped WA powder with spherical powder will increase both flowability and apparent density roughly proportional to the fraction. This offers a first possibility to improve the processability in PBF, if needed.
(3) In-situ alloying of WA iron powder blended with master alloys in the PBF-EB process is feasible. Homogeneous distribution of alloying elements is demonstrated for a steel with low content of alloying elements as well as FeCrAl with 18% Cr and 7.5% Al.

The main impacts of these findings are:

(1) For the PBF-EB process, it appears feasible to replace a substantial amount of spherical powder with water atomized powder. Because of the large price difference between gas atomized and water atomized powder, currently by roughly a factor of ten, this represents an appreciable potential for cost saving. Obviously, the PBF processability of water atomized powders could be improved in several ways. The WA process could be tailored to better suit the required morphology and particle size. The WA powder might be modified, e.g., by milling or mixing with modifiers to improve flowability. Finally, modification to the powder feeding mechanism could improve the quality of spread layers [26].
(2) Blending inexpensive base feedstock with alloying elements offers higher flexibility in additive manufacturing, as there is no need for atomization of each individual alloy composition. While the results of in-situ alloying of WA iron were chemically homogeneous alloys in this work, non-equilibrium microstructures without full alloying are also interesting for future research, especially by adding hard and high melting point phases.

## Figures and Tables

**Table 1:** Tensile properties of the PBF-EB iron/10 wt% 316L/0.25 wt% C specimen.

Condition	YTS	UTS	A
As-built	231 ± 4 MPa	454 ± 12 Mpa	17.5 ± 1.5%
Heat-treated	416 ± 5 Mpa	543 ± 4 Mpa	9.0 ± 1.5%

## Data Availability

The data that support the findings of this study are available from the corresponding author upon reasonable request.
